# Comparison of the discriminative ability of a generic and a condition-specific OHRQoL measure in adolescents with and without normative need for orthodontic treatment

**DOI:** 10.1186/1477-7525-6-64

**Published:** 2008-08-21

**Authors:** Eduardo Bernabé, Cesar M de Oliveira, Aubrey Sheiham

**Affiliations:** 1 Unidad de investigación en Salud Pública Dental, Departamento de Odontología Social, Universidad Peruana Cayetano Heredia, Lima, Perú; 2 Department of Epidemiology and Public Health, University College London, London, UK

## Abstract

**Background:**

At present, there is no evidence on whether using condition-specific Oral Health-Related Quality of Life (OHRQoL) measures provides more reliable information than generic measures for needs assessment. Therefore, the objective was to assess the discriminative ability of one generic and one condition-specific OHRQoL measure, namely, respectively, the short form of the Oral Health Impact Profile (OHIP-14) and the Condition-Specific form of the Oral Impacts on Daily Performances (CS-OIDP) attributed to malocclusion, between adolescents with and without normative need for orthodontic treatment.

**Methods:**

200 16–17-year-old adolescents were randomly selected from 957 schoolchildren attending a Sixth Form College in London, United Kingdom. The impact of their oral conditions on quality of life during the last 6 months was assessed using two OHRQoL measures; OHIP-14 and OIDP. Adolescents were also examined for normative orthodontic treatment need using the Index of Orthodontic Treatment Need (IOTN) and the Dental Aesthetic Index (DAI). Discriminative ability was assessed comparing the overall scores and prevalence of oral impacts, calculated using each OHRQoL measure, between adolescents with and without normative need. Using the prevalence of oral impacts allowed adjusting for covariates.

**Results:**

There were significant differences in overall scores for CS-OIDP attributed to malocclusion between adolescents with and without normative need for orthodontic treatment when IOTN or DAI were used to define need (p = 0.029 or 0.011 respectively), and in overall scores for OHIP-14 when DAI, but not IOTN was used to define need (p = 0.029 and 0.080 respectively). For the prevalence of impacts, only the prevalence of CS-OIDP attributed to malocclusion differed significantly between adolescents with and without normative need, even after adjusting for covariates (p = 0.017 and 0.049 using IOTN and DAI to define need).

**Conclusion:**

CS-OIDP attributed to malocclusion was better able than OHIP-14 to discriminate between adolescents with and without normative needs for orthodontic treatment.

## Background

Oral Health-Related Quality of Life (OHRQoL) can be assessed using either generic or specific measures [1,2]. Generic OHRQoL measures take into account numerous oral conditions, some occurring simultaneously, and thus collect information about wider effects of oral health on daily living. The main advantage of generic measures is that they allow comparison of various domains of quality of life for the condition being studied, as well as across populations and disease states [3-6]. One of the most commonly used generic OHRQoL measures is the two versions of Oral Health Impact Profile (OHIP); with 49 or 14 items [7,8]. On the other hand, specific OHRQoL measures focus on a particular disease, condition, symptom, function or population and thus are used when any of the aforementioned specific attributes needs to be assessed [1,4,5]. Condition-specific instruments are the most commonly used specific OHRQoL measures [1], probably because they provide more information on consequences of a specific untreated oral condition or disease and the corresponding benefits of its treatment [3,6]. The Oral Impacts on Daily Performances (OIDP) is the only OHRQoL measure designed to link specific oral conditions, such as malocclusion, and impacts on quality of life [9,10].

It has been claimed that condition-specific OHRQoL measures may increase acceptability to subjects by including only relevant dimensions [1,3,6]. In addition, their specific focus makes them potentially more sensitive to small, but clinically important changes in oral health [1,4,5]. This may in turn increase responsiveness [1,3], which is particularly important when assessing oral health needs. Knowing whether there is an impact of the mouth on quality of life does not necessarily provide information on what specific dental condition was related to the impact. Condition-specific OHRQoL measures attempt to provide such information by attributing oral impacts to specific oral conditions, therefore indicating which conditions may require dental attention [11]. In this sense, the condition-specific form of the OIDP index (CS-OIDP) is an integral part of the socio-dental approach for oral health needs assessment [12-14].

Although using condition-specific OHRQoL measures for needs assessment seems theoretically sound, some recent studies have also assessed oral health needs using generic OHRQoL measures [15,16]. Empirical evidence may cast light on whether using condition-specific OHRQoL measures provides more reliable information than generic measures. To do that, both types of OHRQoL measures must be evaluated first in terms of their ability to differentiate between groups differing in health statuses. Such an evaluation is part of construct validity assessment [4,17-19]. There is no evidence on whether generic or condition-specific OHRQoL measures are more appropriate for assessing dental needs. Therefore, the objective of this study was to assess the discriminative ability of one generic and one condition-specific OHRQoL measure, namely, respectively, the short form of the Oral Health Impact Profile (OHIP-14) and the Condition-Specific form of the Oral Impacts on Daily Performances (CS-OIDP) attributed to malocclusion, between adolescents with and without normative need for orthodontic treatment.

## Methods

### Population and setting

Two hundred 16–17-year-old adolescents were randomly selected from a list containing the names of all the 957 schoolchildren attending the Havering Sixth Form College in London, United Kingdom during 2006. All the students selected agreed to take part in the study. Sample size was calculated to estimate a prevalence of 25% for the condition-specific oral impacts on daily performances attributed to malocclusion, with a maximum tolerable error of 5% [20].

The Local Ethics Committee and the Research and Development Directorate of the University College London Hospitals National Health Service Trust approved this study. Participants signed a consent letter agreeing for their participation in the study.

### Data collection

First, information about demographic characteristics (sex, age and ethnicity), orthodontic treatment status and the impact of oral conditions on quality of life during the last 6 months was self-reported by the participants. Information about oral impacts was collected using OHIP-14 and OIDP. Adolescents self-completed OHIP-14 in their classrooms and were later interviewed individually with OIDP in a private room. The OHIP-14, which has been previously validated on British populations [21,22], assesses the frequency of problems associated with the mouth, teeth or dentures on 7 dimensions: functional limitation, physical pain, psychological discomfort, physical disability, psychological disability, social disability and handicap. Adolescents were asked to rate each of the 14 items on a 5-point ordinal scale coded 0 'never', 1 'hardly ever', 2 'occasionally', 3 'fairly often' and 4 'very often'. The overall score for OHIP-14 was obtained summing up all responses, thus ranging between 0 and 56 points [8,23]. The OIDP index, which has also been validated on British populations [21,24], assesses serious oral impacts on 8 daily performances namely, eating, speaking, cleaning mouth, relaxing, smiling, studying, emotion and social contact. If an adolescent reported an impact on any of the 8 performances, the frequency of the impact (scale from 1 to 3) and the severity of its effect on daily life (scale from 1 to 3) were scored. If no impact was reported, then a zero score was assigned. Thereafter, adolescents were asked to identify from a list those oral problems that, in their opinion, caused the impact. Only those Condition-Specific Oral Impacts on Daily Performances related to 'bad position of teeth', 'space between teeth', and 'deformity of mouth or face', were considered in the analysis as CS-OIDP attributed to malocclusion [14,25]. Performance scores were estimated by multiplying the corresponding frequency and severity scores. The overall score for the CS-OIDP attributed to malocclusion was the sum of the 8 performance scores (ranging from 0 to 72), multiplied by 100 and divided by 72 [9,10].

Adolescents were then examined for normative orthodontic treatment need using both components of the Index of Orthodontic Treatment Need (IOTN) as well as the Dental Aesthetic Index (DAI). Both indexes have gained international acceptance because they are valid, reliable and easy to use [26-28]. For the Dental Health Component (DHC) of IOTN, 10 traits of malocclusion were assessed: overjet, reverse overjet, overbite, openbite, crossbite, crowding, impeded eruption, defects of cleft lip and palate as well as any craniofacial anomaly, Class II and Class III buccal occlusions, and hypodontia. Only the highest scoring trait is used to assess treatment need [29]. Thereafter, adolescents self-rated their dental attractiveness on the 10-point scale of the Aesthetic Component (AC) of IOTN [29,30]. Results from DHC and AC of IOTN were merged into a single classification according to the current General Dental Services regulations of the National Health Services in United Kingdom [31,32]. According to these regulations, orthodontic care can only be provided for individuals who have a DHC grade of 4 or 5, or grade 3 with an AC of 6 or above. All other cases were therefore classified as having no need. For DAI, 10 occlusal traits were assessed and a score was obtained using the equation: 6×(missing visible teeth) + crowding + spacing + 3×(diastema) + largest anterior maxillary irregularity + largest anterior mandibular irregularity + 2×(anterior maxillary overjet) + 4×(anterior mandibular overjet) + 4×(vertical anterior openbite) + 3×(anteroposterior molar relation) + 13 [33,34]. Each adolescent was then classified as having no need (score < 28) or need (score ≥ 28) [27]. Examinations were carried out by one of the authors (CMO), who had been previously trained and calibrated in the Department of Orthodontics at University of Cardiff where the IOTN was developed. According to weighted Kappa, inter- and intra-examiner reliability were 0.77 and 0.91 respectively.

### Data analysis

Discriminative ability was examined in terms of construct validity whereby the distributions of scores for both OHRQoL measures are compared between groups with different levels of oral health [18]. Since overall scores for OHIP-14 and CS-OIDP attributed to malocclusion were not normally distributed (Shapiro-Wilks test, p < 0.001 in all cases), Mann-Whitney tests were used to compare both overall scores between adolescents with and without normative need for orthodontic treatment. To aid comparison and interpretation, the magnitude of differences was also expressed as an effect size [35,36], which was calculated as the mean difference between groups divided by the pooled standard deviation. The widely accepted thresholds of 0.2, 0.5 and 0.8 were used to define 'small', 'moderate' and 'large' effect sizes [35].

As the aforementioned method did not allow adjusting for covariates (sex, age, ethnicity and orthodontic treatment status), the prevalence of oral impacts was also compared between adolescents with and without normative need for orthodontic treatment. For that, the prevalence of oral impacts was calculated as the percentage of adolescents reporting one or more items 'fairly often' or 'very often' for OHIP-14 [23] and as the percentage of adolescents with a score higher than zero for CS-OIDP attributed to malocclusion [10]. Then, the prevalence of oral impacts was compared between adolescents with and with normative need using Poisson regression with robust estimation of variance while adjusting for covariates [37,38].

## Results

This study included 134 (67.0%) females and 66 (33.0%) males, 116 (58.0%) were aged 16 years and 84 (42.0%) aged 17 years; 170 were Caucasian (85%) and 30 (15.0%) were of other ethnic origins. One third (32.5%) had completed orthodontic treatment, 12.5% were currently undergoing orthodontic treatment and the remaining 55.0% were untreated. Based on the two measures of orthodontic need, 42 (21.0%) had a normative need for orthodontic treatment according to IOTN whereas 25 (12.5%) had a normative need using DAI.

There were significant differences in the overall scores for CS-OIDP attributed to malocclusion between adolescents with and without normative need for orthodontic treatment when IOTN or DAI were used to define need (p = 0.029 or 0.011 respectively), and in the overall scores for OHIP-14 when DAI, but not IOTN was used to define need (p = 0.029 and 0.080 respectively). Using DAI, the mean difference in overall scores for OHIP-14 and CS-OIDP attributed to malocclusion between adolescents with and without normative need was 1.64 points (CI95%: -0.84; 4.12) and 2.13% (CI95%: 0.44; 3.81) respectively. The corresponding size effects for such mean differences in overall scores were 0.28 (CI95%: -0.14; 0.70) and 0.53 (CI95%: 0.11; 0.95) respectively (Table 1). Using IOTN, the mean difference in overall score for CS-OIDP attributed to malocclusion between adolescents with and without normative need was 1.35% (CI95%: -0.03; 2.72) and its corresponding size effect was 0.33 (CI95%: -0.01; 0.68).

**Table 1:** Comparison of the overall score for OHIP-14 and CS-OIDP attributed to malocclusion between adolescents with and without normative need for orthodontic treatment.

**OHRQoL** ** measure**	**Normative** ** need**	**n**	**Mean**	**SD**	**p** value*	**Effect** ** size**	**95% CI for** ** effect size**
OHIP-14	No need by IOTN	158	5.13	6.00	0.080	0.13	(-0.21; 0.47)
(0–56 points)	Need by IOTN	42	5.88	5.49			
	No need by DAI	175	5.08	5.95	0.029	0.28	(-0.14; 0.70)
	Need by DAI	25	6.72	5.37			

CS-OIDP	No need by IOTN	158	1.13	3.63	0.029	0.33	(-0.01; 0.68)
(0–100%)	Need by IOTN	42	2.48	5.25			
	No need by DAI	175	1.15	3.67	0.011	0.53	(0.11; 0.95)
	Need by DAI	25	3.28	5.84			

* Mann-Whitney test was used.

In addition, there were significant differences in the prevalence of oral impacts between adolescents with and without normative need for orthodontic treatment only for CS-OIDP attributed to malocclusion (p = 0.032 and 0.049 respectively), but not for OHIP-14 (p = 0.799 and 0.211 respectively). This finding was independent of the index used to define normative need for orthodontic treatment (Table 2). After adjusting for covariates, adolescents with normative need for orthodontic treatment had respectively an 1.89 (CI95%: 1.12; 3.20) and 1.84-fold (CI95%: 1.00; 3.39) increase in the chance of reporting CS-OIDP attributed to malocclusion, compared to adolescents without normative need, when the IOTN and DAI were used to define need.

**Table 2:** Comparison of the prevalence of oral impacts, by OHIP-14 and CS-OIDP attributed to malocclusion, between adolescents with and without normative need for orthodontic treatment.

		**Prevalence**				
					
**OHRQoL** ** measure**	**Normative** ** need**	**n**	**%**	PR*	**95% CI** ** for PR**	**p** ** value**
OHIP-14	No need by IOTN	21	13.3	1.00		
	Need by IOTN	6	14.3	1.12	(0.50; 2.49)	0.790
	No need by DAI	21	12.0	1.00		
	Need by DAI	6	24.0	1.64	(0.76; 3.55)	0.211

CS-OIDP	No need by IOTN	29	18.4	1.00		
	Need by IOTN	14	33.3	1.89	(1.12; 3.20)	0.017
	No need by DAI	33	18.9	1.00		
	Need by DAI	10	40.0	1.84	(1.00; 3.39)	0.049

* Poisson regression was used to calculate prevalence ratios (PR) adjusted for sex, age, ethnicity and orthodontic treatment status.

## Discussion

This study evaluated two widely used OHRQoL measures, OHIP-14 and CS-OIDP attributed to malocclusion, in terms of their ability to discriminate adolescents with, from those without normative need for orthodontic treatment. This was the first attempt to assess the discriminative ability of both OHRQoL measures.

When overall scores for both OHRQoL measures were used to assess the impacts of oral conditions on everyday life, adolescents with normative need for orthodontic treatment always reported significantly higher OHRQoL scores than adolescents without normative need, except for the OHIP-14 overall score when IOTN was used to define need. One explanation for this finding relates to sample size. As this study was based on secondary analysis of a prevalence study [20], no evaluation of the statistical power for comparison purposes could be done. Though, it must be noted that the group with normative need was smaller when DAI than when IOTN was used to define need (25 versus 42 adolescents), and that there were group differences even with that smaller DAI sample. An alternative explanation may relate to well-known differences between DAI and IOTN [26,39,40]. With IOTN only the worst occlusal trait is recorded, which is not necessarily related to the participant's oral impact. In other words, occlusal traits that affect dental appearance and have an impact on participants' daily lives may not be captured by IOTN. In addition, DAI has many more measures of malocclusion affecting the anterior teeth than the IOTN. For example, DAI includes number of missing visible teeth, crowding in the incisal segments, spacing in the incisal segment, and measurement of any midline diastema that are not specifically addressed by IOTN. However, such differences could not explain why CS-OIDP attributed to malocclusion, but not OHIP-14 differentiated adequately between adolescents with and without normative need as defined by both indexes. Therefore, this finding indicates that the expected more sensitive, condition-specific OHRQoL measure better discriminated between adolescents with and without normative need for orthodontic treatment than the generic OHRQoL measure.

Furthermore, when effect sizes were used to interpret the magnitude of mean differences in scores between adolescents with and without normative need for orthodontic treatment, better results were found for CS-OIDP attributed to malocclusion than for OHIP-14. Effect size for CS-OIDP attributed to malocclusion was moderate whereas effect size for OHIP-14 was nil when DAI was used to define normative need for orthodontic treatment.

When the prevalence of oral impacts, calculated by each OHRQoL measure, was used to assess the impacts of oral conditions on everyday life, differences between adolescents with and without normative need for orthodontic treatment were found for CS-OIDP attributed to malocclusion but not for OHIP-14. This was independent of whether DAI or IOTN was used to define need. Generally, adolescents with normative need for orthodontic treatment had slightly more than four-fifth increase in the probability of reporting CS-OIDP attributed to malocclusions after controlling for the effects of covariates (sex, age, ethnicity and orthodontic treatment status). The comparison of prevalences between groups with different oral health statuses has been reported for other OHRQoL measures [41-43]. Unquestionably, this was an advantage over using mean differences because there is no way to control for covariates with non-parametric tests such as the Mann-Whitney test.

Overall, different findings were found when comparing the discriminative ability of OHIP-14 and CS-OIDP attributed to malocclusion between groups with and without normative need for orthodontic treatment. These findings differed according to the indicator used to assess the impacts of oral conditions on participants' quality of life (the overall score or the prevalence of oral impacts) or the index used to define normative need for orthodontic treatment (IOTN or DAI). However, based on the present findings it appears that CS-OIDP attributed to malocclusion was better able than OHIP-14 to differentiate between the two groups of adolescents based on needs. Therefore, the present findings confirmed our earlier assumption that the condition-specific OHRQoL measures were better able to discriminate between sub-groups with different levels of oral health than their generic counterparts. This also provides empirical support for using condition-specific OHRQoL measures for oral health needs assessment.

Our findings agree with the few previous studies comparing generic and condition-specific OHRQoL measures [42-44]. They showed that both OHRQoL measures are complementary, rather than alternative sources of information. Although this holds true for situations in which researchers are interested in assessing not only the overall profile of oral impacts but also those impacts on quality of life related to specific oral conditions, the present findings raise the important question, does using a generic or a condition-specific OHRQoL measure provide additional information for oral health needs assessment when the specific link between a specific oral condition leading to impacts on quality of life is required to prioritise need for professional attention? The findings from this study suggest that a condition-specific OHRQoL measure should be used in such situations. However, since these findings were based on distinguishing between adolescents with and without a specific type of normative need, they need further confirmation for other oral health needs.

## Conclusion

Among a population of 16–17-year-old British adolescents, the CS-OIDP attributed to malocclusion was better able than the more generic OHIP-14 to discriminate between different levels of normative need for orthodontic treatment. Findings differed according to the indicator used to assess the impacts of oral conditions on participants' quality of life (overall score or prevalence of oral impacts) or the index used to define normative need for orthodontic treatment (IOTN or DAI).

## Competing interests

The authors declare that they have no competing interests.

## Authors' contributions

EB conceived of the study, performed statistical analysis and drafted the first version of the manuscript. CMdO organized and conducted the study, and has critically revised the manuscript. AS supervised the entire study and critically revised the manuscript. All authors read and approved the final version of the manuscript.
